# Absence of apolipoprotein E protects mice from cerebral malaria

**DOI:** 10.1038/srep33615

**Published:** 2016-09-20

**Authors:** Fikregabrail Aberra Kassa, Kristin Van Den Ham, Anthony Rainone, Sylvie Fournier, Eric Boilard, Martin Olivier

**Affiliations:** 1 Department of Medicine, Microbiology and Immunology, Faculty of Medicine, McGill University, Montréal, Québec, Canada; 2 The Research Institute of the McGill University Health Centre and Infectious Diseases and Immunity in Global Health Program, Montréal, Québec, Canada; 3 Centre de Recherche du Centre Hospitalier Universitaire de Québec, Université Laval, Québec, Québec, Canada

## Abstract

Cerebral malaria claims the life of millions of people each year, particularly those of children, and is a major global public health problem. Thus, the identification of novel malaria biomarkers that could be utilized as diagnostic or therapeutic targets is becoming increasingly important. Using a proteomic approach, we previously identified unique biomarkers in the sera of malaria-infected individuals, including apolipoprotein E (ApoE). ApoE is the dominant apolipoprotein in the brain and has been implicated in several neurological disorders; therefore, we were interested in the potential role of ApoE in cerebral malaria. Here we report the first demonstration that cerebral malaria is markedly attenuated in ApoE^−/−^ mice. The protection provided by the absence of ApoE was associated with decreased sequestration of parasites and T cells within the brain, and was determined to be independent from the involvement of ApoE receptors and from the altered lipid metabolism associated with the knock-out mice. Importantly, we demonstrated that treatment of mice with the ApoE antagonist heparin octasaccharide significantly decreased the incidence of cerebral malaria. Overall, our study indicates that the reduction of ApoE could be utilized in the development of therapeutic treatments aimed at mitigating the neuropathology of cerebral malaria.

Cerebral malaria is a neurological complication of malaria that claims the life of millions of people every year, particularly those of children under the age of five^1^. It is mainly characterized by coma and is fatal if left untreated, making it the leading cause of malaria-related death. Pathology is caused by multiple factors, particularly the sequestration of parasitized red blood cells (RBCs) and leukocytes within the brain, which leads to the hallmark disruption of the blood-brain barrier (BBB)^2^,^3^.

Cerebral malaria is currently treated with anti-malarial drugs, such as quinine/quinidine or artemisnin derivatives^4^. These drugs are very effective at eliminating the parasite, but the anti-malarial treatment needs to be initiated during the early stages of the disease. Even with the timely administration of anti-malarial drugs, 20–30% of patients still succumb to cerebral malaria^5^. Once the disease has progressed to severe malaria, patients will receive adjunctive treatments in conjunction with anti-malarial drugs, in an attempt to lessen the associated pathophysiological processes^5^. However, only a few adjunctive therapies have shown potential in reducing morbidity^4^,^5^. Therefore, there is a growing need for the identification of novel mechanistic players to develop effective adjunctive therapies.

Based on this, we previously utilized a proteomic approach to discover unique biomarkers in the sera of malaria patients that could potentially be used as therapeutic targets^6^. One of the identified biomarkers was apolipoprotein A (ApoE), a 34 kDa glycoprotein that is produced by hepatic parenchymal cells and astrocytes, and that is abundant in the plasma^6^,^7^. ApoE is known to influence the onset and the rate of progression of several neurological disorders such as Alzheimer’s disease, cerebral amyloid angiopathy and traumatic brain injury^7^,^8^,^9^. In Alzheimer’s disease, ApoE was shown to modulate pathology by altering the aggregation and clearance of amyloid-β from the brain, and by modifying neuroinflammation and brain lipid transport^10^,^11^.

Furthermore, ApoE has also been demonstrated to play a role in malaria infection. It was reported that ApoE is able to inhibit the infection of liver cells by malaria sporozoites by interacting with *heparan sulfate proteoglycans* receptors, which are utilized by the parasite to enter the host hepatocytes^12^. Additionally, ApoE is also able to interact with parasite surface proteins, particularly the early transcribed membrane proteins^13^. Notably, *APOE* polymorphisms have been associated with an increased risk of developing severe malaria^13^,^14^. Therefore, we were interested in elucidating whether ApoE played a role in the development of cerebral malaria pathology. Herein we report that ApoE^−/−^ mice are significantly protected against cerebral malaria, and that pharmacological inhibition of ApoE using heparin octasaccharide (OCTA) could represent a new avenue in the development of adjunctive therapies for cerebral malaria.

## Results

### ApoE deletion protects mice from cerebral malaria

**Figure 1:**
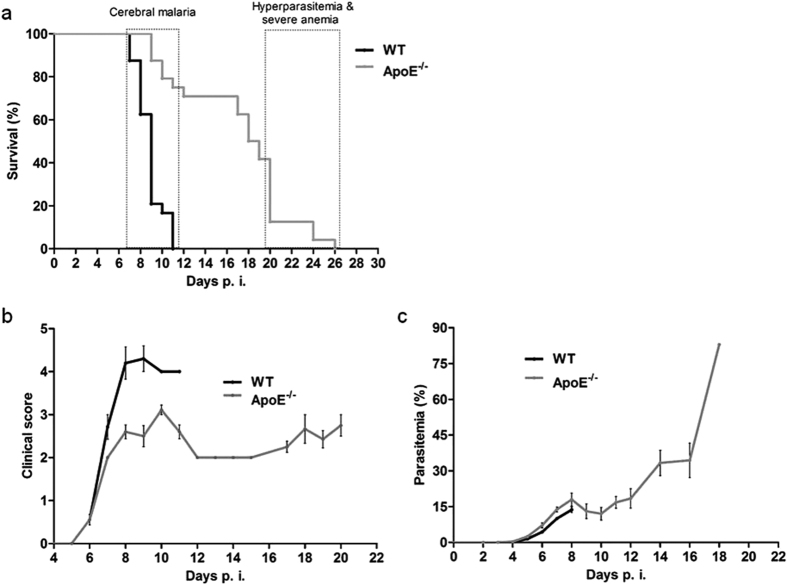
ApoE^−/−^ mice are resistant to cerebral malaria. (**A**) Survival curve of ApoE^−/−^ and WT mice. Log-rank *P* < 0.0001. (**B**) Clinical score of ApoE^−/−^ and WT mice infected with *P. berghei* ANKA. (**C**) Parasitemia curve of ApoE^−/−^ and WT mice infected with *P. berghei* ANKA. Three independent experiments are shown. *n* = 24 for WT mice and *n* = 24 for ApoE^−/−^ mice.

We utilized the experimental cerebral malaria (ECM) model to investigate cerebral malaria in a laboratory setting. In ECM, C57BL/6 mice are infected with *Plasmodium berghei* ANKA and develop symptoms characteristic of cerebral malaria, such as limb paralysis, convulsions and coma, before succumbing to the infection. We sought to evaluate the role of ApoE in ECM using ApoE knock-out mice^15^. The absence of the ApoE glycoprotein in the serum was confirmed by western blot (Supplementary Fig. S1). The wild-type (WT) mice succumbed to the disease within 7 to 11 days post-infection, whereas the ApoE^−/−^ mice were significantly protected, and survived into the fourth week of infection (Fig. 1A). By day 11 post-infection, all of the WT mice had succumbed to the infection, compared to only 25% of the ApoE^−/−^ mice. The median survival time was 9 days post-infection for the WT mice and 18 days post-infection for the ApoE^−/−^ mice. Furthermore, the WT mice displayed the characteristic features of cerebral malaria, but the ApoE^−/−^ mice that survived the cerebral phase did not display these symptoms (Fig. 1B). The non-symptomatic ApoE^−/−^ mice developed hyperparasitemia; the level of parasitemia in the *P. berghei* ANKA-infected WT mice only reached 15–20%, but the ApoE^−/−^ mice that did not develop ECM had a parasitemia as high as 70–80% (Fig. 1C). This hyperparasitemia, and the consequent anemia, appear to be responsible for the eventual death of the ApoE^−/−^ mice^16^.

### ApoE^−/−^ mice have a significantly lower parasite burden in the brain

**Figure 2:**
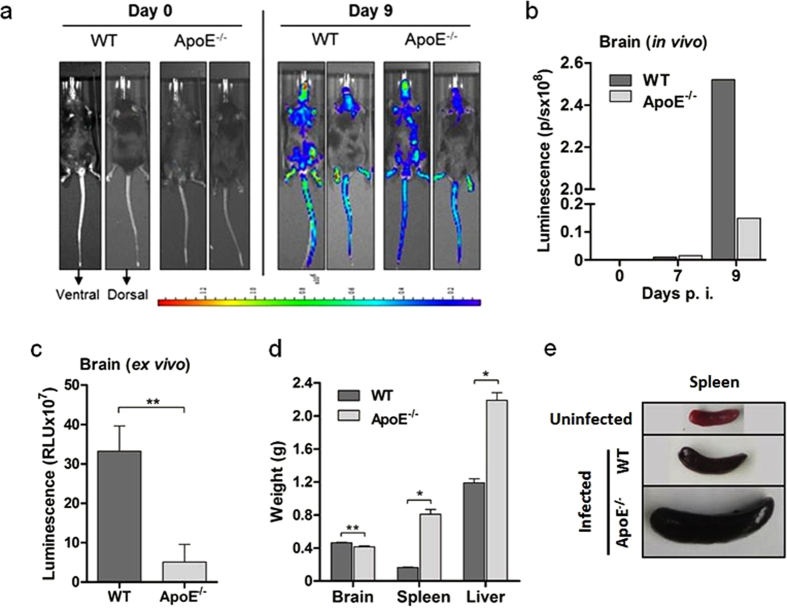
ApoE^−/−^ mice have a significantly lower parasite burden in the brain. (**A**) Visualization and (**B**) quantification of brain luciferase activity in ApoE^−/−^ and WT mice using *in vivo* imaging. (**C**) Luciferase activity of infected WT and ApoE^−/−^ mice on day 9 post-infection assessed *ex vivo. n* = 5 for WT mice and *n* = 5 for ApoE^−/−^ mice; *P* = 0.0070. (**D**) Weight of brain, spleen and liver in infected WT (day 9 post-infection) and ApoE^−/−^ mice (day 24 post-infection). For the brain: *n* = 5 for WT mice and *n* = 5 for ApoE^−/−^ mice, *P* = 0.0022; for the spleen: *n* = 6 for WT mice and *n* = 3 for ApoE^−/−^ mice, *P* = 0.0238; and for the liver: *n* = 6 for WT mice and *n* = 3 for ApoE^−/−^ mice, *P* = 0.0238. (**E**) Representative pictures of spleens from WT (no infection and day 9 post-infection) and ApoE^−/−^ mice (day 24 post-infection). **P ≤ 0.01 and ***P ≤ 0.0001.

One of the major characteristics of ECM is the accumulation of malaria parasites within the brain^17^. The sequestration of pRBC and leukocytes in the brain is associated with immunopathological changes in the brain microvasculature, including hemorrhage and edema, and the subsequent development of cerebral symptoms, leading to death^18^. We assessed the level of parasite sequestration in the brain of the infected WT and ApoE^−/−^ mice using live *in vivo* imaging. To quantify the parasite burden, we used transgenic *P. berghei* ANKA_GFP-LUC_ that constitutively express luciferase. Parasite accumulation in the brain and whole body was measured *in vivo* using bioluminescence on day 9 post-infection (Fig. 2A). The sequestration of parasites in the brain was significantly lower in the ApoE^−/−^ mice compared to the WT mice (Fig. 2B). Additionally, the brains of the infected WT and ApoE^−/−^ mice were extracted and assessed for parasite burden using a luciferase assay. The parasite burden in the brain was also shown to be significantly lower in the ApoE^−/−^ mice compared to the WT mice using this method (Fig. 2C). Total brain weight was used as a measure of edema. The brains from of the ApoE^−/−^ mice weighed significantly less than the brains of the WT mice, suggesting that the ApoE^−/−^ mice may have had less edema (Fig. 2D). The weight of the spleen and liver in the ApoE^−/−^ mice was similar to the WT mice at the onset of symptoms, and significantly increased throughout the duration infection, likely due to the infiltration of parasitized RBCs and the deposition of hemozoin (Fig. 2D, Supplementary Fig. S2). In particular, the spleen, which is the principal site for the clearance of parasitized RBCs^19^,^20^, was approximately four times larger in the ApoE^−/−^ mice by the end of the infection (Fig. 2D,E).

### Deletion of ApoE prevents the disruption of the BBB

**Figure 3:**
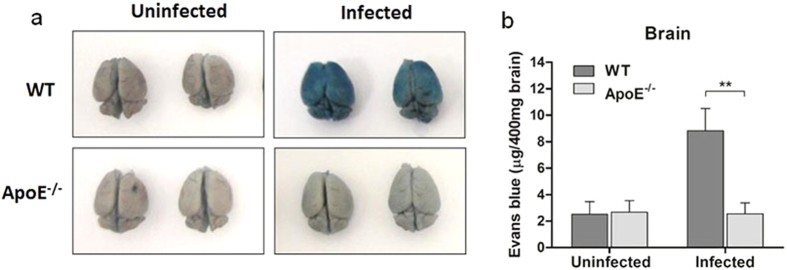
ApoE^−/−^ mice infected with *P. berghei* ANKA maintain BBB integrity. (**A**) Representative pictures of brains from uninfected and infected WT and ApoE^−/−^ mice injected with Evans blue. (**B**) Quantification of Evans blue in the brain parenchyma. *n* = 6 for uninfected and infected WT mice and *n* = 8 for uninfected and infected ApoE^−/−^ mice; *P* = 0.0035. Infected WT mice analyzed on day 9 post-infection and infected ApoE^−/−^ mice analyzed on day 16 post-infection (based on their median survival times). **P ≤ 0.01.

BBB disruption is a hallmark of cerebral malaria pathology. The tight junctions between the endothelial cells in the BBB restrict the passage of large molecules and pathogens, and maintain the interface between the blood and the brain^3^. During cerebral malaria, the integrity of the BBB is compromised, leading to hemorrhage and coma^3^,^21^. The integrity of the BBB in the infected WT and ApoE^−/−^ mice was assessed using Evans blue^21^. The brains of the infected WT mice were stained intensely by Evans blue, indicating a widespread increase in vascular permeability and rupture of the BBB (Fig. 3A). Contrastingly, there was only a small amount of dye leakage in the ApoE^−/−^ mice, similar to that of the uninfected controls (Fig. 3A). The inhibited extravasation of Evans blue into the brain was quantified by measuring the amount of Evans blue extracted from the brains using formamide (Fig. 3B). The amount of Evans blue in the blood was measured in the same manner to confirm that Evans blue uptake was equal in all the mice (Supplementary Fig. S3).

### ApoE^−/−^ mice have significantly decreased infiltration of T cells into the brain

**Figure 4:**
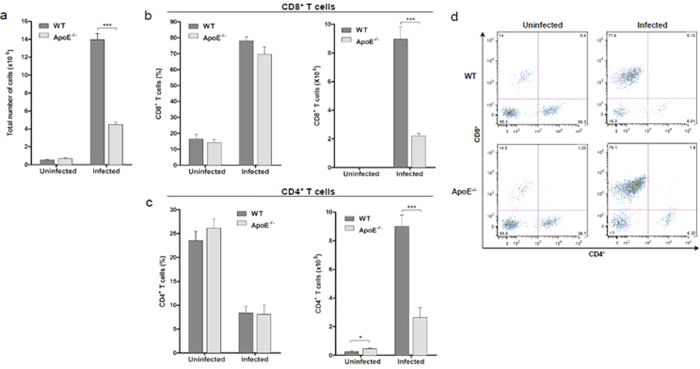
Sequestration of CD4^+^ and CD8^+^ T cells in the brain is decreased in ApoE^−/−^ mice. (**A**) Total number of cells accumulated within the brain, *P* < 0.0001. (**B**) Percentage and total number of CD8^+^ T cells in the brain, *P* < 0.0001. (**C**) Percentage and total number of CD4^+^ T cells in the brain, *P* = 0.0364. (**D**) Representative flow cytometric dot plots of CD4^+^ and CD8^+^ T cells sequestered within the brain, after gating on infiltrating leukocytes (CD45^+^ CD11b^lo-hi^). Percentage of CD4^+^ and CD8^+^ T cells calculated as a percentage of infiltrating leukocytes. *n* = 5 for uninfected WT and ApoE^−/−^ mice, *n* = 6 for infected WT mice and *n* = 7 for infected ApoE^−/−^ mice. *P ≤ 0.05 and ***P ≤ 0.0001. Infected, WT and ApoE^−/−^ mice were analyzed on day 7 to 9 post-infection (upon the development of clinical symptoms in the WT mice).

Accumulation of T cells in the brain has been shown to contribute to the pathogenesis of ECM. Previous work has determined that infiltration of CD8^+^ T cells in the brain is required for the onset ECM and that CD4^+^ T cells promote the development of neurological symptoms by enhancing the sequestration of CD8^+^ T cells within the brain^22^,^23^,^24^. In order to assess the accumulation of host immune cells in the brains of the infected WT and ApoE^−/−^ mice, the total number and percentage of CD4^+^ and CD8^+^ T cells was determined using flow cytometry. We observed a significant reduction in the total number of immune cells that were recruited to the brain of the infected ApoE^−/−^ mice (Fig. 4A). Most importantly, the accumulation of CD4^+^ and CD8^+^ T cells in the brain was significantly decreased in the infected ApoE^−/−^ mice compared to the infected WT mice (Fig. 4B,C). The absence of ApoE^−/−^ had no effect on the percentage of CD4^+^ or CD8^+^ T cells that sequestered in the brain (Fig. 4B–D). Thus, the decreased accumulation of CD4^+^ and CD8^+^ T cells in the brains of the infected ApoE^−/−^ mice concurs with their increased resistance to ECM pathology.

### ApoE receptors do not play a role in the protection provided by ApoE deletion

**Figure 5:**
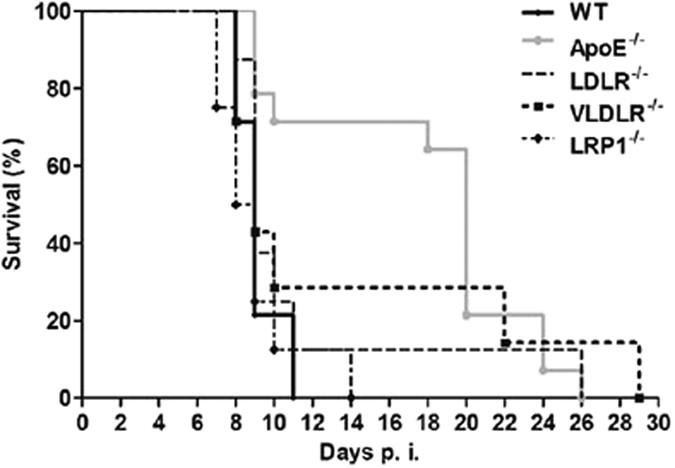
The protection provided by ApoE deletion is independent of its receptors. Survival curve of WT, ApoE^−/−,^ LDLR^−/−^, VLDLR^−/−^ and LRP1^−/−^ mice. Log-rank WT vs. ApoE^−/−^ *P* < 0.0001; WT vs. LDLR^−/−^ *P* = 0.3527; WT vs. VLDLR^−/−^ *P* = 0.2791; and WT vs. LRP1^−/−^ *P* = 0.6349. *n* = 14 for WT mice, *n* = 14 for ApoE^−/−^ mice, *n* = 8 for LDLR^−/−^ mice, *n* = 7 for VLDLR^−/−^ mice, and *n* = 8 for LRP1^−/−^ mice.

ApoE receptors have been shown to mediate intracellular signalling in neurons, and to play a role in the pathogenesis of Alzheimer’s disease^25^. Thus, we were interested in determining whether the resistance of ApoE^−/−^ mice to ECM was mediated through ApoE receptors. We examined the impact of the deletion of three major ApoE receptors, low-density lipoprotein receptor (LDLR), very low-density lipoprotein receptor (VLDLR) and LDL-related receptor 1 (LRP1), on the incidence of disease. The receptor knock-out mice were not significantly protected from developing ECM compared to the WT mice (Fig. 5), which led us to conclude that the ApoE receptors do not contribute to ApoE-mediated ECM pathogenesis.

### Protection against cerebral malaria in the ApoE^−/−^ mice is not due to lipid alterations

**Figure 6:**
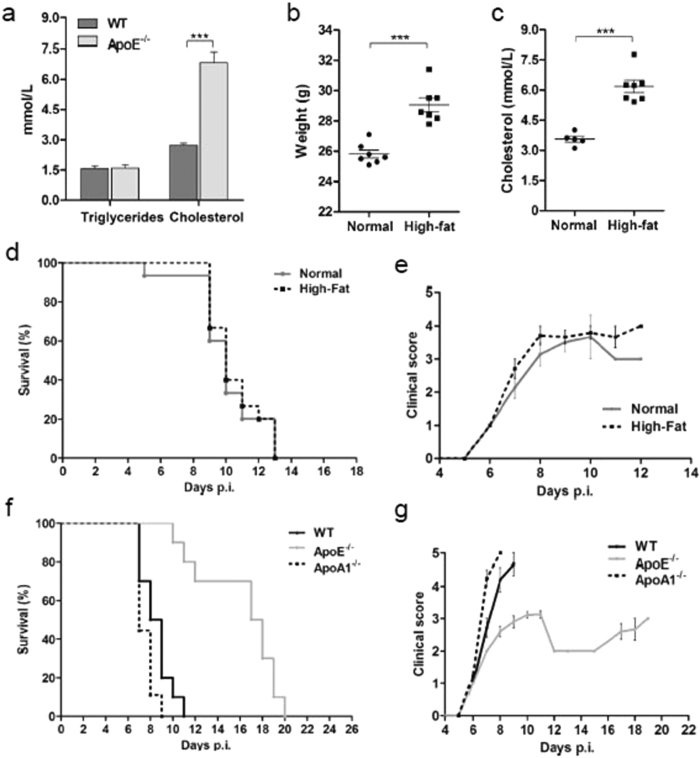
Altered levels of serum lipids in ApoE^−/−^ mice do not contribute to protection. (**A**) Serum triglyceride and total cholesterol levels in uninfected ApoE^−/−^ and WT mice. *n.* = 5 for all groups, *P* < 0.0001. (**B**) Weight of mice fed a standard diet (normal) and a high-fat diet. *n* = 7 for both groups, *P* < 0.0001. (**C**) Serum total cholesterol levels of mice fed a standard diet and a high-fat diet. *n* = 5 for mice fed standard diet and *n* = 7 for mice fed high-fat diet, *P* < 0.0001. (**D**) Survival curve of mice fed a standard diet and mice fed a high-fat diet. Log-rank standard diet vs. high-fat diet Log-rank *P* = 0.09866. *n* = 15 for both groups. (**E**) Clinical scores of mice fed a standard diet and mice fed a high-fat diet. (**F**) Survival curve of WT, ApoE^−/−^ and ApoA1^−/−^ mice. Log-rank WT vs. ApoA1 *P* = 0.0621; WT vs. ApoE^−/−^ *P* < 0.0001; and ApoE^−/−^ vs. ApoA1^−/−^ *P* < 0.0001. *n* = 10 for WT mice, *n* = 10 for ApoE^−/−^ mice, and *n* = 9 for ApoA1^−/−^ mice. (**G**) Clinical score of WT, ApoE^−/−^ and ApoA1^−/−^ mice infected with *P. berghei* ANKA. ***P ≤ 0.0001.

ApoE^−/−^ mice have relatively normal plasma triglyceride levels, but have significantly elevated levels of total cholesterol compared to WT mice^26^. We confirmed these lipid measurements in our own experimental set-up in the uninfected WT and ApoE^−/−^ mice. As expected, the ApoE^−/−^ mice had similar triglyceride levels and significantly increased total cholesterol levels compared to the WT mice (Fig. 6A). Furthermore, the ApoE^−/−^ mice were shown to have significantly reduced levels of high-density lipoprotein (HDL) cholesterol (data not shown). Therefore, we investigated whether the protection against cerebral malaria in the ApoE^−/−^ mice was due to the elevated total cholesterol or the reduced HDL cholesterol. To mimic the increased total cholesterol in the ApoE^−/−^ mice, WT mice were fed a high-fat diet. Body weight and total cholesterol were significantly elevated in the mice fed the high-fat diet compared to the mice fed the standard chow (Fig. 6B,C). We observed that the mice fed a high-fat diet were as susceptible to cerebral malaria as the mice fed the standard chow (Fig. 6D,E), suggesting that the elevated total cholesterol in the ApoE^−/−^ mice does not contribute to the protective phenotype. ApoA1^−/−^ mice, which have significantly decreased levels of HDL cholesterol compared to WT mice^27^, were used to determine if the reduced HDL cholesterol contributed to the protective phenotype of the ApoE^−/−^ mice. The incidence of ECM was not significantly decreased in the ApoA1^−/−^ mice compared to the WT mice, suggesting that the decreased level of HDL cholesterol is not responsible for the protection against cerebral malaria observed in the ApoE^−/−^ mice (Fig. 6F,G). Overall, we determined that neither elevated total cholesterol nor reduced HDL cholesterol levels in the ApoE^−/−^ mice are responsible for protection.

### Platelet depletion, deletion of the serotonin transporter and fluoxetine (Prozac) treatment partially protect mice from cerebral malaria

Sequestration of platelets in the brain is thought to contribute to the pathology of ECM by augmenting the accumulation of leukocytes and pRBCs, contributing to immune-mediated cytotoxicity, and by obstructing cerebral capillaries^28^. Platelet depletion has been shown to protect mice from ECM, and ApoE has been reported to antagonize platelet function by inhibiting platelet aggregation^29^,^30^,^31^. We depleted platelets using anti-CD42 antibodies, to determine whether thrombocytopenia protected mice from cerebral malaria. The platelet-depleted mice were determined to be significantly more protected from cerebral malaria compared to the IgG–injected controls (Supplementary Fig. S4A).

Furthermore, platelet-derived microparticles and serotonin have also been shown to be important mediators of platelet function. Microparticles originating from platelets have been shown to bind to parasitized RBCs, leading to cytoadherence and obstruction of capillaries^29^,^32^. Platelet-derived serotonin has been reported to be involved in vasculature leakage in a mouse model of joint inflammation; however, the role of serotonin in cerebral malaria has never been investigated^33^. Platelets do not synthesize serotonin, but contain the vast majority of circulating serotonin through the use of the serotonin transporter (SERT)^34^. Since SERT is also expressed in neurons, we used irradiated C57BL/6 mice that were engrafted with bone marrow from SERT deficient mice to specifically evaluate the contribution of platelet-derived serotonin^33^. The SERT^−/−^ mice were significantly more protected from ECM than the WT controls (Supplementary Fig. S4B). The role of serotonin in ECM pathology was further investigated by treating mice with Prozac, a selective SERT inhibitor. Prozac treatment did not prevent the development of neuropathology, but did delay the onset of ECM (Supplementary Fig. S4C). Thus, platelets and platelet-derived serotonin contribute to the development of ECM; however, they likely do not contribute to the protection observed in ApoE^−/−^ mice, because the ApoE protein itself has been shown to inhibit platelet aggregation.

### ApoE^−/−^ mice have elevated levels of serum proinflammatory and anti-inflammatory cytokines

**Figure 7:**
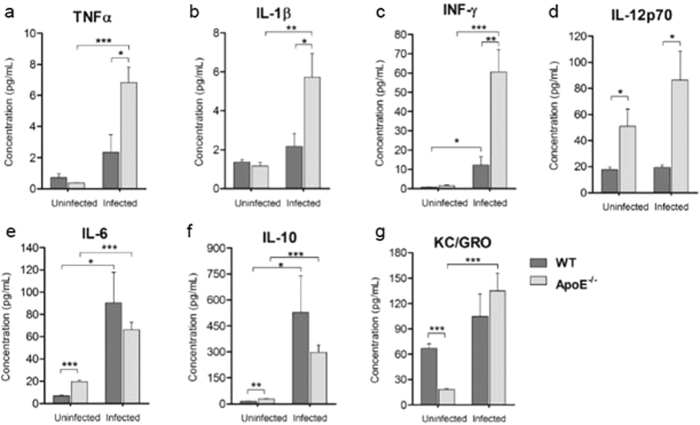
ApoE^−/−^ mice have modulated levels of serum pro-inflammatory and anti-inflammatory cytokines. Serum concentrations of (**A**) TNF-α, uninfected ApoE^−/−^ vs. infected ApoE^−/−^ *P* = 0.0002, infected WT vs. infected ApoE^−/−^ *P* = 0.0166; (**B**) IL-1β, uninfected ApoE^−/−^ vs. infected ApoE^−/−^ *P* = 0.0064, infected WT vs. infected ApoE^−/−^ *P* = 0.0349; (**C**) IFN-γ, uninfected WT vs. infected WT *P* = 0.0241, uninfected ApoE^−/−^ vs. infected ApoE^−/−^ *P* = 0.0009, infected WT vs. infected ApoE^−/−^ *P* = 0.0042; (**D**) IL-12p70, uninfected WT vs. uninfected ApoE^−/−^ *P* = 0.0386, infected WT vs. infected ApoE^−/−^ *P* = 0.0166; (**E**) IL-6, uninfected WT vs. uninfected ApoE^−/−^ *P* < 0.0001, uninfected WT vs. infected WT *P* = 0.0155, uninfected ApoE^−/−^ vs. infected ApoE^−/−^ *P* = 0.0002; (**F**) IL-10, uninfected WT vs. uninfected ApoE^−/−^ *P* = 0.0017, uninfected WT vs. infected WT *P* = 0.0412, uninfected ApoE^−/−^ vs. infected ApoE^−/−^ *P* = 0.0002; and (**G**) KC/GRO, uninfected WT vs. uninfected ApoE^−/−^ *P* < 0.0001, uninfected ApoE^−/−^ vs. infected ApoE^−/−^ *P* = 0.0005 from uninfected and infected WT and ApoE^−/−^ mice. *n* = 5 for all groups. *P ≤ 0.05, **P ≤ 0.01, and ***P ≤ 0.0001. Infected, WT and ApoE^−/−^ mice were analyzed on day 7 to 9 post-infection (upon the development of clinical symptoms in the WT mice).

The pathogenesis of cerebral malaria in mice has an immunopathological component and the serum levels of inflammatory cytokines have been used as important indicators of the progression of cerebral malaria^35^. Therefore, we assessed whether uninfected and infected WT and ApoE^−/−^ mice had differences in the concentration of proinflammatory and anti-inflammatory cytokines. Mice that have ECM symptoms have been shown to produce excessive amounts of proinflammatory cytokines, and ApoE^−/−^ mice were found to have significantly elevated serum levels of these proinflammatory cytokines (Fig. 7A–C). IL-12p70, which is the active heterodimer of IL-12 and stimulates the secretion of IFN-γ^36^, was also significantly increased in the infected ApoE^−/−^ mice (Fig. 7D). IL-6; IL-10, an anti-inflammatory cytokine; and keratinocyte chemoattractant (KC), a neutrophil chemoattractant; were determined to be expressed at similar levels in the infected WT and ApoE^−/−^ mice (Fig. 7E–G). In addition to systemic cytokine production, we also examined the splenic CD4^+^ and CD8^+^ T cells. T cell sequestration within the brain was markedly decreased in the ApoE^−/−^ mice and T cell activation occurs in the spleen during ECM^37^, thus the activation and chemotactic capacity of these cells was examined to determine how the deletion of ApoE was preventing their accumulation within the brain. The chemokine receptor CXCR3 is crucial to the migration of T cells to the brain and the induction of neuropathology^38^; however, no differences in the expression of CXCR3 on splenic CD4^+^ and CD8^+^ T cells were measured in the ApoE^−/−^ mice compared to the WT mice (Supplementary Fig. S5A,B). Furthermore, the activation of CD4^+^ and CD8^+^ T cells was not affected by the deletion of ApoE (Supplementary Fig. S5C,D). Overall, ApoE^−/−^ mice had increased production of proinflammatory cytokines during ECM, but the activation and expression of CXCR3 on splenic T cells was not significantly different.

### OCTA protects mice from cerebral malaria

We clearly demonstrated that ApoE^−/−^ mice were protected from ECM pathology; thus, we were interested in identifying compounds that could be used to antagonize the expression of ApoE in a clinical setting. We hypothesized that OCTA, an oligomeric by-product of the deaminative cleavage of unfractionated heparin, could be a potential candidate. OCTA has been shown to antagonize ApoE by binding to the LDLR-binding region of ApoE^39^, but has not previously been examined as a treatment for cerebral malaria.

**Figure 8:**
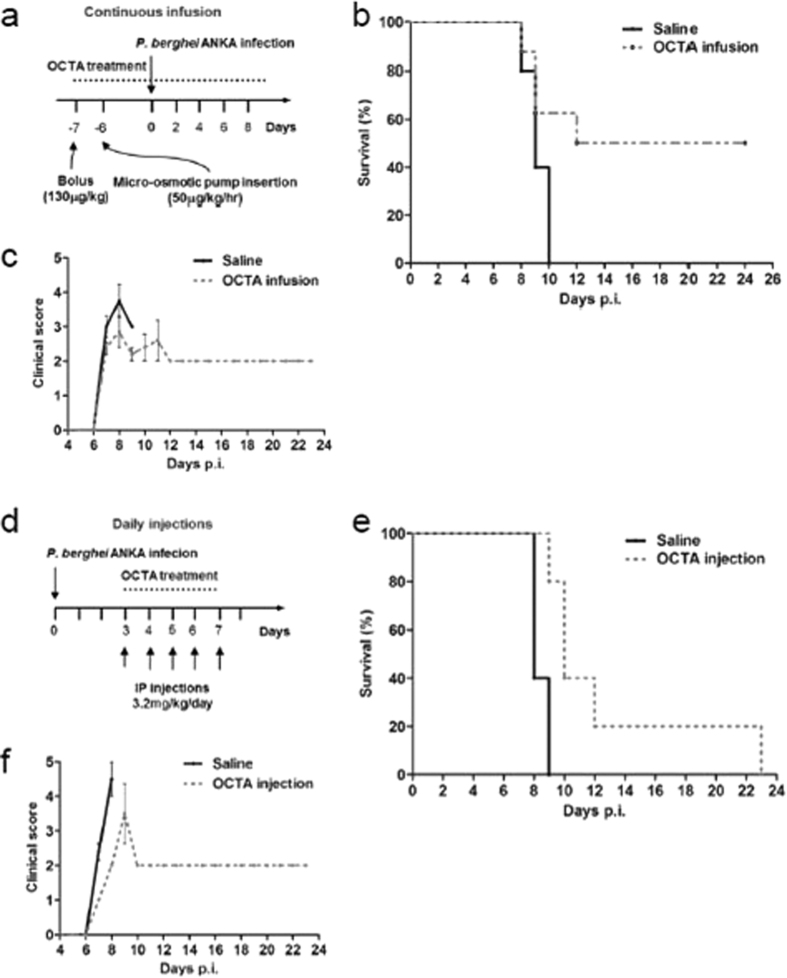
ApoE antagonist OCTA protects mice from cerebral malaria. (**A**) Treatment regime for the continuous infusion of OCTA (**B**) Survival curve for mice infused with saline and mice infused with OCTA. Log-rank saline infusion vs. OCTA infusion *P* = 0.0645. *n* = 5 for saline-infused mice and *n* = 8 for OCTA-infused mice. (**C**) Clinical score of mice infused with saline and mice infused with OCTA. (**D**) Treatment regime for the injection of OCTA. (**E**) Survival curve for mice injected with saline and mice injected with OCTA. Log-rank of saline-injected mice vs. OCTA-injected mice *P* = 0.0080. *n* = 5 for both saline- and OCTA-injected mice. (**F**) Clinical score of mice injected with saline and mice injected with OCTA.

We assessed the use of OCTA as a treatment using two routes of administration: continuous infusion and daily injections. For the infusion, WT mice were given an initial bolus, followed by insertion of a micro-osmotic pump into the abdominal cavity to achieve a continuous infusion of OCTA (Fig. 8A). The saline-infused control mice displayed the clinical symptoms of cerebral malaria and succumbed to the infection, whereas the OCTA-infused mice did not display the clinical symptoms of cerebral malaria and had a cerebral malaria incidence of less than 50% (Fig. 8B,C). For the daily injections, mice were injected daily with OCTA from day 3 to 7 post-infection (Fig. 8D). The OCTA injections were found to significantly protect the mice from cerebral malaria compared to the saline-injected mice (Fig. 8E,F). The parasitemia for the OCTA-infused and daily injection mice was similar to the ApoE^−/−^ mice: the parasitemia was not significantly different compared to the to the saline-treated mice until the control mice succumbed to ECM, and the surviving OCTA-treated mice developed hyperparasitemia (data not shown). These findings demonstrate that OCTA confers protection against cerebral malaria and could potentially be utilized in adjunctive therapies.

## Discussion

We report the first demonstration that the absence of ApoE protects mice from cerebral malaria. ApoE is the major apolipoprotein in the brain and has been implicated in the pathology of several neurological disorders, such as Alzheimer’s disease and cerebral amyloid angiopathy^8^,^40^. Furthermore, ATP-binding cassette transporter A1 (ABCA1) deficient mice were found to have greatly decreased levels of ApoE, and the ABCA1^−/−^ mice were also shown to be markedly protected from developing ECM^40^,^41^,^42^. Thus, we were interested in determining the specific impact of ApoE deficiency on cerebral malaria.

We have established that ApoE is essential for the development of ECM and that the absence of this protein protects mice from malaria-associated neuropathology. 100% of the WT mice displayed the characteristic symptoms of cerebral malaria and succumbed to the infection, whereas the ApoE^−/−^ mice were significantly protected, with an ECM incidence of only 25%. The ApoE^−/−^ mice had a median survival time of 18 days post-infection, compared to only 9 days post-infection for the WT mice. Moreover, the hazard ratio for the infection was 13.02, indicating that the ApoE^−/−^ mice were 13 times less susceptible to ECM than the WT mice.

The surviving ApoE^−/−^ mice did not display any of the hallmark clinical symptoms of cerebral malaria, such as limb paralysis, convulsions and coma. However, severe malaria has two common manifestations: cerebral malaria and severe malarial anemia. Although the ApoE^−/−^ mice were significantly resistant to ECM, their parasitemia increased significantly and they eventually succumbed to hyperparasitemia and severe anemia. Up to 75% of the circulating RBCs were observed to be infected with the malaria parasite in the non-symptomatic ApoE^−/−^ mice by the end of the infection. The high parasite burden resulted in extreme splenomegaly, since the spleen is the principal site for the clearance and sequestration of parasitized RBCs during malaria^19^,^20^.

BBB disruption is a hallmark feature of ECM. Structural and functional changes in the BBB have been shown to be responsible for the clinical symptoms in cerebral malaria patients^43^,^44^. Loss of BBB integrity has also been demonstrated to contribute to the neuropathology observed in mice infected with *P. berghei* ANKA^3^,^21^,^45^. Disruption of the BBB was markedly decreased in the ApoE^−/−^ mice compared to the WT mice; the brains of the ApoE^−/−^ mice were significantly less stained by Evans blue than the brains of the WT mice. The prevention of BBB disruption in the ApoE^−/−^ mice was paramount to their increased resistance to ECM.

Breakdown of the BBB is associated with increased sequestration of parasitized RBCs and CD4^+^ and CD8^+^ T cells in the brain^17^,^22^,^23^. Notably, previous studies have shown that brain-infiltrating, CD8^+^ T cells play a crucial role in the development of ECM pathogenesis, by both contributing directly to the breakdown of the BBB and by enhancing the sequestration of parasitized RBCs within the brain^22^,^46^,^47^. The CD8^+^ T cells contribute to the disruption of the BBB through direct cytotoxicity against endothelial cells^22^. The parasite burden in the brains of the infected ApoE^−/−^ mice was shown to be significantly decreased compared to the infected WT mice, using both live *in vivo* imaging and *ex vivo* analysis. Furthermore, the accumulation of CD4^+^ and CD8^+^ T cells within the brain was also determined to be markedly reduced in the ApoE^−/−^ mice. Thus, the increased resistance of the ApoE^−/−^ mice to ECM correlated with significantly decreased sequestration of parasitized RBCs and T cells within the brain, and this reduced accumulation likely contributed to lack of BBB disruption observed in the ApoE^−/−^ mice.

The ApoE receptors have been shown to contribute to pathogenesis in other diseases^25^; therefore we were interested in determining if the increased survival of the ApoE^−/−^ mice was caused by the lack of interaction of the protein with its receptors in the brain. The potential impact of the ApoE receptors on the decreased incidence of ECM in the ApoE^−/−^ mice was examined by infecting LDLR^−/−^, VLDLR^−/−^ and LRP1^−/−^ mice with *P. berghei* ANKA. None of the receptor knock-out mice that we analyzed were found to be significantly protected from ECM compared to the WT mice, indicating that the protection provided by the deletion of ApoE is independent of the ApoE brain receptors.

In addition to modulating intracellular signalling in the brain by interacting with the receptors, ApoE also plays a significant role in lipid metabolism. Deletion of ApoE is associated with increased levels of total cholesterol and decreased levels HDL cholesterol^26^. Therefore, we investigated whether this change in the lipid profile contributed to the enhanced protection from cerebral malaria observed in the ApoE^−/−^ mice. The potential contribution to the protective phenotype provided by the increased total cholesterol was tested by using a high-fat diet, and the potential role of deceased HDL levels was investigated using ApoA1^−/−^ mice. Neither the high-fat diet mice nor the ApoA1^−/−^ mice demonstrated a reduced incidence of ECM compared to the WT mice, suggesting that the changes in lipid metabolism mediated by the deletion of ApoE do not contribute to the increased resistance of ApoE^−/−^ mice to ECM. Overall, the above results demonstrate that it is the absence of the ApoE glycoprotein itself that is responsible for the protection, rather than the downstream effects of the lack of ApoE on its receptors or on the serum lipid levels.

ApoE has also been shown to have the capacity to inhibit platelet aggregation: both ApoE-containing HDL particles and purified ApoE have been shown to suppress aggregation^30^,^31^. ApoE was determined to inhibit platelet aggregation by stimulating NO synthase and the production of NO by platelets^30^. We demonstrated that the depletion of platelets or the attenuation of platelet-derived serotonin was able to partially protect from ECM; however, since the ApoE protein itself was shown to inhibit platelet activity, it is unlikely that mitigation of platelets contributes to the protection afforded by the deletion of ApoE.

Excessive production of proinflammatory cytokines at late time points in the infection correlates with the breakdown of the BBB and the onset of ECM^35^. However, early production of certain inflammatory cytokines, such as IFN-γ and IL-12, has been associated with protection from ECM in mice, and higher serum levels of IL-12 were found in children with mild malaria compared to children with severe forms of malaria^48^,^49^,^50^. Previously, ApoE^−/−^ mice were shown to upregulate TNF-α, IFN-γ and IL-12 to a greater extent than WT mice after lipopolysaccharide challenge^51^. We observed increased systemic levels of TNF-α, IL-1β, IFN-γ and IL-12p70 in the infected mice ApoE^−/−^ mice compared to the infected WT mice. Importantly, the ApoE^−/−^ mice also had increased levels of IL-12p70 without infection. IL-12was depleted in the ApoE^−/−^ mice throughout infection with *P. berghei* ANKA to determine if the increased levels of this cytokine contributed to protection, but the incidence of ECM was not significantly altered in the IL-12-depleted ApoE^−/−^ mice (data not shown). In addition to cytokine production, we also examined the expression of CXCR3 and the activation of splenic CD4^+^ and CD8^+^ T cells to investigate the cause of the decreased sequestration of T cells within the brain. Neither the activation nor the chemotactic capacity of the splenic T cells was affected by the deletion of ApoE. The expression of CCR5, another chemokine receptor that has been shown to be important in the chemotaxis of T cells to the brain in ECM, was not measured^52^. Thus, it is possible that the reduced accumulation of T cells within the brain of the ApoE^−/−^ mice could be caused by attenuated expression of CCR5.

After establishing that deletion of ApoE inhibited the development of ECM, we were interested in identifying compounds that could be used to antagonize ApoE, and that could potentially be used in the development of adjunctive therapies. Currently, the primary treatment for severe malaria is anti-malarial drugs, such as quinine/quinidine and artemisinin derivatives^4^; however, death still occurs in 10–25% of cerebral malaria cases. Thus, there is a need to develop adjunctive therapies to treat the pathophysiological processes caused by malaria or to mitigate the end-stage factors associated with mortality.

We decided to investigate antagonists that directly target ApoE, since our previous experiments indicated that it is likely the ApoE protein itself, rather than signalling through the ApoE receptors or changes in the lipid profile, that is responsible for the protection in the ApoE^−/−^ mice. Previous studies have demonstrated that heparin is an ApoE antagonist and has the capacity to be used as a treatment for cerebral malaria^39^,^53^,^54^,^55^,^56^,^57^. Heparin was shown to alleviate intracranial pressure and to improve microcirculation, but it is not currently recommended for the treatment of cerebral malaria, since the use of heparin can result in heparin-induced thrombocytopenia, and increases the risk of intracerebral hemorrhage^58^. However, it is plausible to hypothesize that using heparin as a treatment for cerebral malaria would be beneficial if the anti-coagulant properties could be minimized.

Heparin-binding sites are present in both the N- and C-terminal domains of ApoE and the high-affinity heparin binding site in the N-terminal domain overlaps with the LDL receptor-binding region^39^,^54^,^55^. OCTA has been shown to bind to this LDL receptor binding site with comparable affinity to the full-length heparin, and it has been shown to have negligible anticoagulant effects^39^,^53^,^59^. Furthermore, it was demonstrated that OCTA treatment protects mice against septic mortality to almost the same extent as full-length heparin by antagonizing ApoE^53^. Thus, we were interested in investigating the potential of OCTA to be utilized as a treatment for cerebral malaria.

Here we report for the first time that administration of OCTA significantly protects mice from cerebral malaria pathology. The efficacy of OCTA as a treatment was assessed using both daily injections and continuous infusion via a micro-osmotic pump. The incidence of ECM was 20% and the average onset of cerebral malaria was delayed by two days in the mice given daily injections, and the incidence of ECM was 50% in the OCTA-infused mice. Both routes of OCTA administration provided substantial protection from ECM, suggesting that heparin-mediated ApoE antagonism is promising as a treatment for cerebral malaria.

Overall, we have clearly demonstrated that ApoE is essential to the development of cerebral malaria. The protection from ECM provided by the deletion of ApoE correlated with decreased sequestration of parasitized RBCs and T cells within the brain and was independent from the involvement of ApoE receptors and from the altered lipid metabolism present in the knock-out mice. Most importantly, we have demonstrated that the treatment of mice with the ApoE antagonist OCTA significantly decreases the incidence of ECM and that OCTA has the potential to be used in the development of novel cerebral malaria adjunctive therapies.

## Materials and Methods

### Animals and parasites

ApoE^−/−^, LDLR^−/−^ and VLDLR^−/−^ mice were a generous gift from Dr. Nabil Seidah (IRCM). ApoA1^−/−^ mice were generous gift from Dr. Sylvia Vidal (McGill University) and SERT^−/−^ mice were obtained from Dr. Eric Boilard (Laval University). LRP1^−/−^ mice were purchased from Jackson Laboratories. All mice were bred and maintained under conventional conditions. We used the malaria parasite *P. berghei* ANKA line 676m1cl1 (MRA-868, MR4, ATCC Manassas Virginia), which express GFP-luciferase constitutively throughout their life cycle. This parasite line was obtained from the Malaria Research and Reference Reagent Resource Center (MR4). Mice received intraperitoneal injections of 1 × 10^4^ infected RBCs. Animals were checked twice daily for the development of neurological symptoms. A clinical score from 0 to 5 was employed as follows: 0 = normal, active; 1 = ruffled fur, less active; 2 = panting, coat staring; 3 = hunched, wobbly gait; 4 = limb paralysis, immobile and 5 = convulsions and coma. Scores 4 and 5 are characteristic features of cerebral malaria in susceptible mice infected with *P. berghei* ANKA. Parasitemia was determined by using blood samples collected from the tail and blood smears stained with Diff-Quick (Siemens). All research involving mice was carried out in accordance with the regulations of the Canadian Council of Animal Care and was approved by the McGill University Animal Care Committee under ethics protocol number 5925 and the Research Institute of the McGill University Health Centre Animal Care Committee under ethics protocol number 7607. Mice were euthanized at established humane endpoints using CO_2_ asphyxiation followed by cervical dislocation or by using isoflurane if perfusion was performed.

### *In vivo* visualization and quantification of luciferase activity

Visualization and quantification of luciferase activity in whole bodies or isolated brains of *P. berghei* ANKA infected and uninfected WT and ApoE^−/−^ mice was achieved using an intensified-charge-coupled device (I-CCD) video camera and *in vivo* Imaging System (IVIS 100, Xenogen)^60^. The mice were anesthetized by using isofluorane (Baxter), shaved and were injected intraperitoneally with 5 mg/ml D-Luciferin (Caliper Life Sciences). Measurements were performed within 3 to 5 min after the injection of D-luciferin. Luciferase-expressing regions were visualized using the *in vivo* Imaging system. The mice were euthanized and the brains were placed in a Petri dish and imaged. The bioluminescence was quantified by selecting a region of interest and using average radiance (p/s/cm^2^/sr) as the unit.

### *Ex vivo* brain luciferase activity

Brains from infected WT and ApoE^−/−^ mice were weighed and then homogenized in *Firefly* luciferase lysis buffer (Biotium, Hayward CA). The luciferase assay was performed on the lysate as per the manufacturer’s (Biotium) instructions. The luminescence was measured using a luminescence plate reader.

### Blood–brain barrier permeability assessment

BBB permeability assessed using the Evans blue assay^20^. Uninfected and infected WT and ApoE^−/−^ mice were injected intravenously with 200 μL of 2% Evans blue dye at the onset of the neurological phase (Sigma-Aldrich). After one hour, the mice were sacrificed and the brains were weighed and placed in formamide (Sigma-Aldrich) for 48 h. The optical density of the extracted dye was measured at 610 nm.

### Flow cytometry analysis

Mice were perfused for the analysis of brain sequestered cells. Brains were digested in RPMI containing collagenase (1.6 mg/mL; type IV; Sigma Aldrich) and DNaseI (200 μg/mL; Sigma Aldrich) at 37 °C for 50 min. Cells were isolated using a Percoll gradient (GE Healthcare). Debris was filtered out using a 70 μm nylon mesh. Cells were counted and labelled with LIVE/DEAD amine-reactive violet viability maker according to the manufacturer’s protocol (Invitrogen). The cells were blocked and labeled with FITC anti-CD45 (eBioscience; 30-F11), PE anti-CD11b (BD Pharmingen; M1/70), APC anti-CD4 (eBioscience; RM4-5) and PerCP-Cy5.5 anti-CD8 (eBioscience; 53–6.7). Flow cytometry was performed using a BD LSR Fortessa and results were analyzed using the FACS Diva 6.0 software.

### High-fat diet experiments

Mice were fed either standard chow or a high-fat diet chow for 21 days prior to infection with *P. berghei* ANKA. The standard chow consists of 18% protein and 5% fat, with 16% calories coming from fat (2920X, Harlan Teklad, Madison, WI). The high-fat diet consists of 17.3% protein, 21.2% fat and 0.2% cholesterol, with 42% calories coming from fat (TD.88317, Harlan Teklad, Madison, WI). After three weeks, mice were infected with *P. berghei* ANKA and their survival, clinical signs and total body weights were monitored. Blood samples were collected for serum lipid analysis of cholesterol levels.

### Serum lipid analysis

For the determination of serum concentrations of cholesterol and triglycerides, blood samples were collected by cardiac puncture from uninfected and infected WT and ApoE^−/−^ mice. For the high-fat diet experiments, blood was also collected by cardiac puncture from mice fed the high-fat diet or standard chow diet. Serum samples were analyzed by the Automated Chemical Analyzer (Vitros 250 Chemistry System, Johnson & Johnson) at the McGill University Animal Clinical Laboratory core facility.

### Analysis of serum cytokine concentrations

Serum from uninfected and *P. berghei* ANKA infected WT and ApoE^−/−^ mice was isolated as outlined above and serum concentrations of IFN-γ, IL-1β, IL-6, IL-10, IL-12p70, KC/GRO/CINC, and TNF-α were quantified using the Mouse ProInflammatory 7-Plex Ultra-Sensitive Kit (Meso Scale, Gaithersburg, MD), according to the manufacturer’s protocol. The plates were analyzed by a SECTOR Imager 2400 plate reader (Meso Scale).

### OCTA treatments

The use of OCTA as a treatment for cerebral malaria was assessed using two routes of administration: continuous infusion and daily injections. For the infusion, WT mice were given an initial bolus (500 μL) of either saline or 130 μg/kg OCTA (Neoparin, Alameda, CA) intraperitoneally one week prior to infection with *P. berghei* ANKA. The day after the bolus, the mice were anaesthetized using isoflurane and a small midline incision was made in the skin below the rib cage, followed by a small incision in the abdominal muscle. A micro-osmotic pump (ALZET, Durect Corporation, Cupertino, CA) containing 100 μL of saline or OCTA was inserted into the peritoneal cavity^49^ and the skin incision was closed with AutoClip & Reflex wound closure (Durect Corporation). The micro-osmotic pumps continuously infused OCTA at a rate of 50 μg/kg/hr for four weeks. For the administration of OCTA via intraperitoneal injections, WT mice were infected with *P. berghei* ANKA and on days 3–7 post-infection, 3.2 mg/kg of OCTA or saline. OCTA-treated and saline-treated control mice were monitored for mortality and clinical symptoms of cerebral malaria as outlined above.

### Statistical analysis

Statistical differences for survival curves were analysed by Log-Rank. Statistical differences between groups were analysed using an unpaired two-tailed student’s t-test or the Mann-Whitney U test, and data were expressed as the mean ± standard error of the mean (SEM). *P* values ≤ 0.05 were considered to be significant. All statistical analyses were performed using GraphPad Prism 5.

## Additional Information

**How to cite this article**: Kassa, F. A. *et al.* Absence of apolipoprotein E protects mice from cerebral malaria. *Sci. Rep.* **6**, 33615; doi: 10.1038/srep33615 (2016).

## Supplementary Material

Supplementary Information
