# Supplementary material for: Absence of apolipoprotein E protects mice from cerebral malaria
Source: Sci Rep. 2016 Sep 20;6:33615. doi: 10.1038/srep33615 (PMC5028887; doi:10.1038/srep33615)
Supplement: Supplementary Information [file srep33615-s1.pdf]

Absence of apolipoprotein E protects mice from cerebral malaria

Fikregabrail Aberra Kassa,[1,2] Kristin Van Den Ham,[1,2]
Anthony Rainone,[1] Sylvie Fournier,[1] Eric Boilard,[3] and Martin Olivier[1,2,*]

[1]Department of Medicine, Microbiology and Immunology, Faculty of Medicine, McGill University, Montréal, Québec, Canada; [2]The Research Institute of the McGill University Health Centre and Infectious Diseases and Immunity in Global Health Program, Montréal, Québec, Canada; [3]Centre de Recherche du Centre Hospitalier Universitaire de Québec, Université Laval, Québec, Québec, Canada

*Corresponding author:

Martin Olivier, Ph.D.
1001 boul. Décarie
Site Glen, Block E
Montréal, Québec, H4A 3J1, Canada
Email: martin.olivier@mcgill.ca

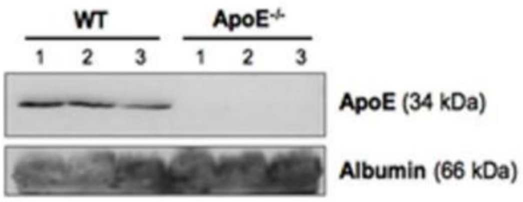

Supplementary Figure S1. ApoE glycoprotein absent from the serum of ApoE$^{-/-}$ mice. Western blot of ApoE in WT and ApoE$^{-/-}$ mice. *n* = 3 from WT and ApoE$^{-/-}$ mice.

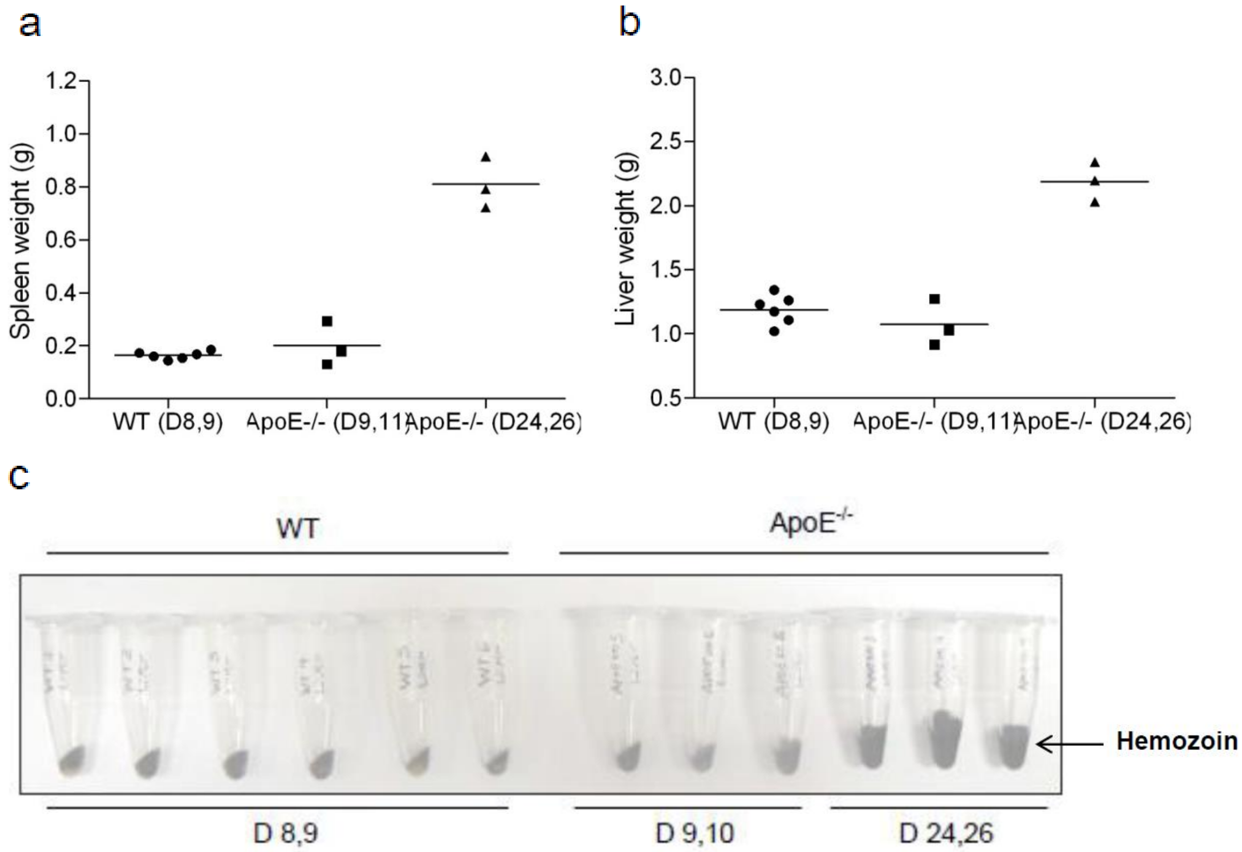

Supplementary Figure S2. Weight of the spleen and liver, and liver deposition of hemozoin in infected mice. (**A**) Graph of spleen weight. (**B**) Graph of liver weight. (**C**) Representative figures of hemozoin accumulation in the livers of WT (day 8 and 9 post-infection) and ApoE$^{-/-}$ (day 9 and 10, and day 24 and 26 post-infection) mice. *n* = 6 for WT mice on day 8 and 9 pos-infection, *n* = 3 for ApoE$^{-/-}$ mice on day 9 and 10 post-infection, and *n* = 3 for ApoE$^{-/-}$ mice on day 24 and 26 post-infection.

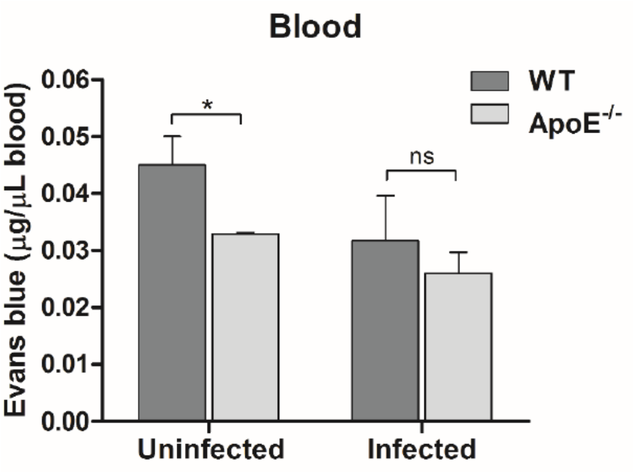

Supplementary Figure S3. Uptake of Evans blue into the blood. Quantification of Evans blue in the blood. *n* = 2 for uninfected WT mice, *n* = 3 for uninfected ApoE$^{-/-}$ mice, *n* = 6 for infected WT mice, and *n* = 8 for infected ApoE$^{-/-}$ mice; *P* = 0.0475 for uninfected mice and *P* = 0.4894 for infected mice. Infected WT mice analyzed on day 9 post-infection and infected ApoE$^{-/-}$ mice analyzed on day 16 post-infection, (based on their median survival times). ns $P \geq 0.05$ and * $P \leq 0.05$.

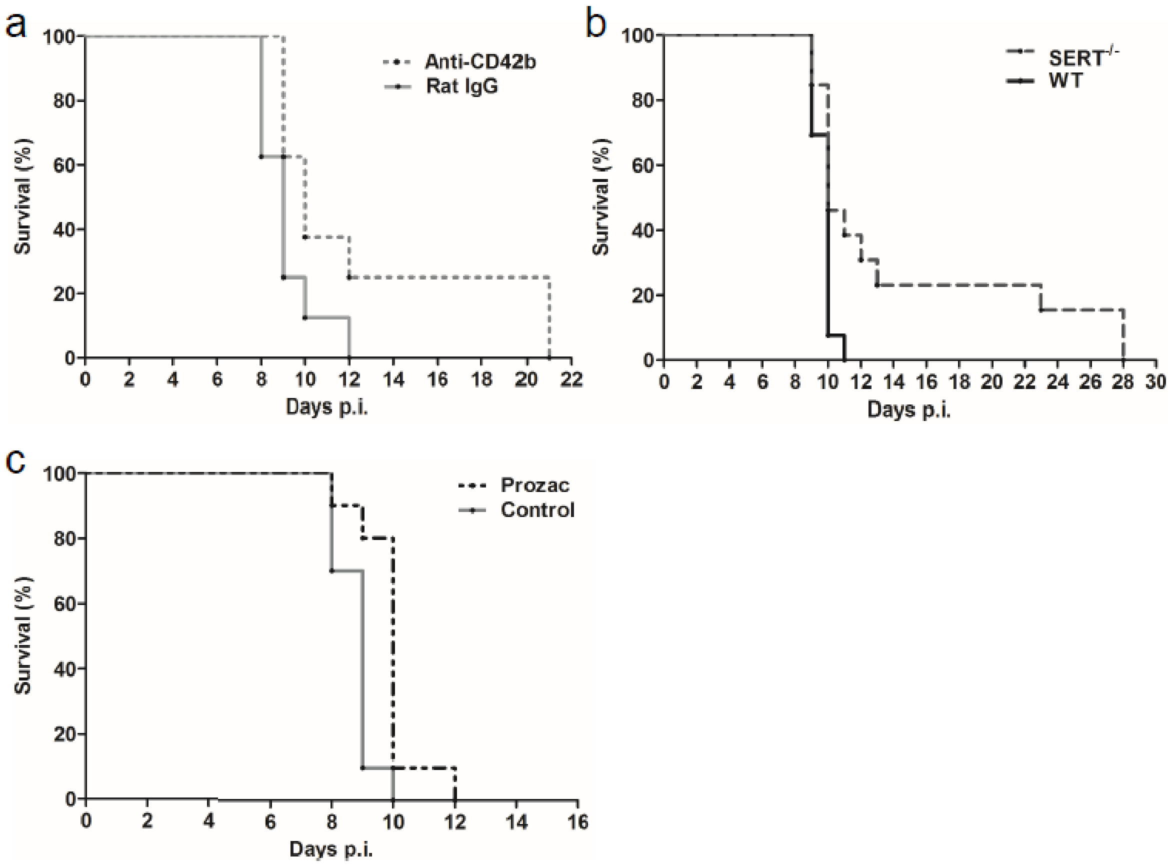

Supplementary Figure 4. Platelet depletion, deletion of SERT and Prozac treatment partially protect mice from cerebral malaria. (**A**) Survival curve of control (Rat IgG) mice and mice treated with anti-CD42b. Log-rank P = 0.0524. n = 8 for the anti-CD42b-treated mice and n = 8 for the control mice. (**B**) Survival curve of WT and SERT$^{-/-}$ mice. Log-rank P = 0.0163. n = 13 for the WT mice and n = 13 for the SERT$^{-/-}$ mice. (C) Survival curve of control mice and mice treated with Prozac. Log-rank P = 0.0040. n = 10 for the control mice and n = 10 for the Prozac-treated mice.

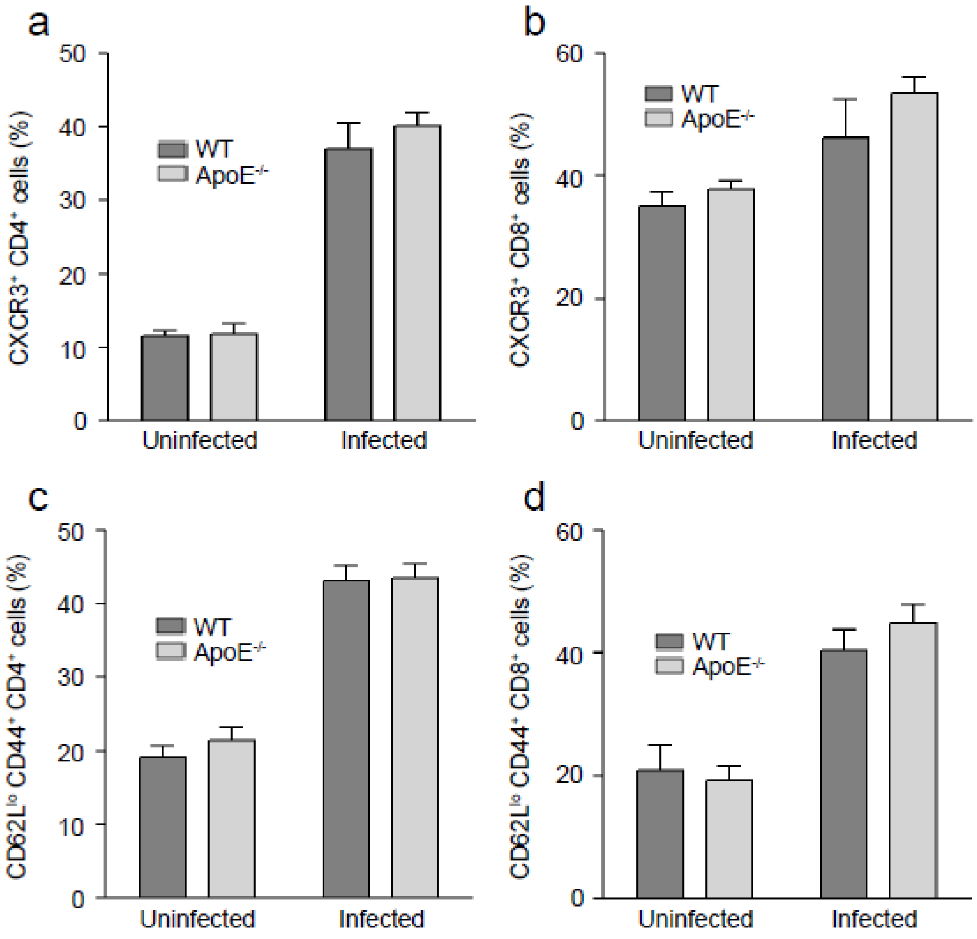

Supplementary Figure S5. CXCR3 expression and activation of splenic CD4$^+$ and CD8$^+$ T cells is not affected by ApoE deletion. (**A**) Percentage of CXCR3$^+$ cells after gating on CD4$^+$ T cells. (**B**) Percentage of CXCR3$^+$ cells after gating on CD8$^+$ T cells. (**C**) Percentage of CD62L^{lo}CD44$^+$ cells after gating on CD4$^+$ T cells. (**D**) Percentage of CD62L^{lo}CD44$^+$ cells after gating on CD8$^+$ T cells. All experiments were performed upon the onset of ECM symptoms in the infected, WT mice. *n* = 7 for the uninfected WT and ApoE$^{-/-}$ mice, and *n* = 10 for the infected, WT and ApoE$^{-/-}$ mice.

SUPPLEMENTARY MATERIALS AND METHODS

Western blot of ApoE and albumin.

SDS-polyacrylamide gel resolved proteins were transferred to PVDF membranes. After blocking, membranes were immunoblotted with Apo E (Millipore) and albumin (Santa Cruz Biotechnology) antibodies and appropriate secondary antibody conjugated with HRP (Sigma).

Quantification of hemozoin deposition.

Equal portions of liver tissue were homogenized in PBS using a PRO200 Hand-held Homogenizer (Harvard Apparatus Canada). The tissue homogenate was sonicated for 5 min, followed by centrifugation at 13,000 rpm for 5 min. Sonication and centrifugation was repeated five times.

Assessment of Evans blue uptake.

Uninfected and infected WT and $ApoE^{-/-}$ mice were injected intravenously with 200 μL of 2% Evans blue dye at the onset of the neurological phase (Sigma-Aldrich). After one hour, the mice were sacrificed and the blood collected by cardiac puncture. The optical density of the dye was measured at 610 nm.

Platelet depletion, SERT and Prozac treatment. Platelet depletion was achieved using a monoclonal antibody directed against mouse CD42b. WT mice were injected intraperitoneally with 0.1 mg anti-CD42b antibody (Emfret Analytics) on day 4 post-infection. IgG–injected mice were used as control. For SERT experiments, mice lacking the SERT and WT mice were infected with *P. berghei* ANKA and survival was monitored as above. For Prozac treatment, WT mice were treated with Prozac, a selective serotonin re-uptake inhibitor. The contents of 20 mg Prozac pills were dissolved in 125 mL of drinking water[31]. Mice were treated for 3 weeks prior

to infection and the treatment continued after infection until the end of the experiment. The drinking water with Prozac was changed once a week and survival was monitored as above.

Flow cytometry analysis of splenic CD4$^+$ and CD8$^+$ T cells.

Flow cytometry was performed using a BD LSR Fortessa and results were analyzed using FlowJo version 9.6.2. Splenocytes were isolated and erythrocytes were lysed in Tris-NH_4Cl buffer. Cells were counted, blocked with and labelled with PerCP-Cy5.5 anti-CD4 (BD Pharmingen; RM4-5), APC-eFluor780 anti-CD8 (eBioscience; 53-6.7), APC anti-CXCR3 (BioLegend; CXCR3-173), FITC anti-CD62L (eBioscience; MEL-14), and PE-Cy7 anti-CD44 (BD Pharmingen; IM7).
